# Circadian nutrition meets intermittent hypoxia: a timely fix for dysglycemia?

**DOI:** 10.1093/sleep/zsaf403

**Published:** 2025-12-18

**Authors:** Hadas Masury, Shirel Fradkov, Alex Gileles-Hillel

**Affiliations:** The Wohl Institute for Translational Medicine, Hadassah Medical Center, Jerusalem, Israel; The Wohl Institute for Translational Medicine, Hadassah Medical Center, Jerusalem, Israel; Faculty of Medicine, Hebrew University of Jerusalem, Jerusalem, Israel; The Wohl Institute for Translational Medicine, Hadassah Medical Center, Jerusalem, Israel; Faculty of Medicine, Hebrew University of Jerusalem, Jerusalem, Israel; Pediatric Pulmonology and Sleep Unit, Department of Pediatrics, Hadassah Medical Center, Jerusalem, Israel

Obstructive sleep apnea (OSA) and type 2 diabetes mellitus are highly comorbid conditions, complicating each other’s management. Despite recent advances in our understanding of these two diseases, the treatment of OSA-induced dysglycemia and insulin resistance remains challenging. Continuous positive airway pressure therapy has an inconsistent effect on metabolic dysfunction in OSA [1, 2], and even the newer Glucagon-Like Peptide-1 (GLP-1) agonist medications often fail to fully normalize these abnormalities [3], underscoring an urgent need to find novel approaches to this difficult clinical scenario.

In this issue of the journal, Afif et al. [4] investigated the effects of time restricted eating (TRE) as one such approach, examining various metabolic endpoints in a murine model of OSA, using a combination of high-fat diet (HFD) and chronic intermittent hypoxia (IH). Collectively, the study shows that the impaired metabolic environment created by IH, in particular, IH-induced dysglycemia and dyslipidemia, responds distinctly to TRE. Fasting glucose levels declined significantly over time exclusively in the IH–TRE group. In terms of glucose tolerance, ad libitum (ad lib) feeding progressively worsened glycemic responses throughout the exposure period, while TRE improved glucose excursions in both room air controls and IH animals. Interestingly, IH–ad lib mice displayed unexpectedly low circulating insulin relative to their hyperglycemia, suggesting either an insufficient insulin response or a failure of pancreatic β-cell secretion. This finding implicates the pancreas as a crucial site of pathology and supports the hypothesis that TRE’s benefits are derived from enhanced pancreatic endocrine function. To test this hypothesis, the authors examined several markers of pancreatic function. In the IH–ad lib group, they found an elevated serum proinsulin-to-insulin ratio, an indicator of β-cell processing failure, with TRE normalizing this ratio. This functional restoration occurred despite a TRE-induced general reduction in pancreatic β-cell mass across all groups, suggesting that the benefit was mechanistic rather than structural. The authors also examined miR-152-3p, a microRNA that negatively regulates genes involved in insulin synthesis and secretion. It was elevated in IH–ad lib mice but normalized by TRE in the IH group. This normalization coincided with favorable changes in the mRNA level of several pancreatic enzymes within the islets: TRE increased glucokinase, which senses glucose in the β-cell, and prohormone convertase 1/3 (PC1/3), the enzyme critical for efficient proinsulin-to-insulin conversion. These results support a model in which TRE corrects IH-induced glucose defects by uniquely optimizing insulin synthesis and secretion machinery.

The results of this study support some previous investigations demonstrating that IH directly impairs pancreatic β-cell function. Chronic IH exposure has been associated with reduced glucose-stimulated insulin secretion, increased β-cell apoptosis, and disruption of key regulatory pathways involved in insulin biosynthesis [5, 6]. In a rat IH model, pretreatment with the antioxidant *N*-acetylcysteine significantly attenuated β-cell death, implicating IH-induced oxidative stress as the culprit [7]. Collectively, these findings suggest that reduced β-cell survival and function play a role in the development of type 2 diabetes observed in patients with OSA.

Why does eating at certain time windows have such beneficial metabolic effects? TRE is a form of intermittent fasting that has emerged as a dietary strategy to reduce body weight and improve glycemic control. It restricts food intake to a specified daily window, typically 4–10 h, followed by water-only fasting for the remainder of the day. TRE has been associated with modest weight loss, typically ranging from 1 per cent to 4 per cent of baseline body weight, largely due to the reduced energy intake that results from restricting eating hours [8]. TRE is an effective weight-loss strategy for individuals with overweight or obesity. In individuals with prediabetes, it also seems to lower fasting insulin levels, increase insulin sensitivity, and improve glucose tolerance [8].

In one study of early 6-h TRE in men with obesity and prediabetes, body weight remained stable, yet participants experienced gains in insulin sensitivity, beta-cell function, and blood pressure [9]. These results resonate with those from the Afif et al. study, whereby both IH and TRE led to reduced total body mass and adiposity, but only TRE improved glycemic control, indicating that its metabolic benefits in IH are not simply a consequence of weight or fat loss.

Since mice are nocturnal animals, TRE is typically scheduled during their active phase, ensuring that feeding aligns with their natural circadian behavior [10]. Importantly, this paradigm differs from classical caloric restriction because the total caloric intake is kept equal to ad libitum controls, making the timing of feeding, rather than the amount, the primary variable under investigation [11]. In the Afif et al. study, food intake was lower in the TRE group, but the difference between TRE and ad lib controls was minimal in the IH group, suggesting that the TRE benefits observed in the IH group may also reflect circadian feeding rather than purely caloric restriction. Circadian feeding refers to the way the body’s internal clock regulates daily rhythms in food intake and metabolism. The master clock in the suprachiasmatic nucleus synchronizes peripheral clocks in metabolic tissues, and while light is the main cue for the central clock, feeding time strongly influences peripheral clocks, especially in the liver and gastrointestinal tract. Normally, eating occurs during the active phase, which supports efficient glucose tolerance, insulin secretion, and energy use. When this timing is disrupted, through eating during the rest phase, shift work, or irregular meal patterns, circadian desynchronization occurs, increasing the risk of obesity, impaired glucose tolerance, and metabolic syndrome [12]. OSA has also been associated with circadian misalignment [13], potentially driving increased eating of carbohydrate- and fat-rich foods during the day [14, 15] and the downstream maladaptive metabolic effects.

In a recent murine study, TRE produced strong metabolic benefits, including reduced adiposity, improved glucose tolerance, greater insulin sensitivity, and protection against hepatic steatosis and inflammation, even when caloric intake matched that of ad libitum controls. These effects resulted from improved alignment of peripheral circadian clocks during active-phase feeding, and broad circadian-driven gene expression changes across metabolic tissues [16].

Disappointingly, isocaloric TRE in humans does not yield similar metabolic improvements. Isocaloric trials show no significant effects on insulin sensitivity, glucose, lipids, blood pressure, or body composition, despite high adherence. TRE does shift circadian clock gene expression and sleep timing [17], but in contrast to the animal data, these circadian changes do not produce measurable metabolic benefits when calories remain constant [18, 19].

An important limitation of the current study is the model used. Mice were fed HFD and subsequently exposed to IH and TRE. This model is particularly relevant, as obesity commonly coexists with OSA in humans. Nevertheless, since the effects of TRE under normal diet conditions remain poorly characterized, it is important to determine whether the observed outcomes are specific to this metabolic context or can also be recapitulated in a normal-diet model, which would more accurately reflect non-obese individuals with OSA. Furthermore, although the pancreatic insulin response was examined, peripheral insulin resistance was not. It is well established that OSA induces, in part through chronic inflammation and oxidative stress, insulin resistance in peripheral organs such as adipose tissue and muscle [20, 21]. It would be important to understand if TRE also has effects on this pathway, in addition to its beneficial effects on the pancreas.

In summary, the study by Afif et al. [4] demonstrated that TRE has beneficial metabolic effects in IH mice with diet-induced obesity, potentially through improved pancreatic insulin secretion. This novel study opens an avenue for the adoption of such an approach in human OSA, where the current therapies are insufficient to mitigate the maladaptive metabolic response, bearing in mind the limitations of the isocaloric approach as delineated above.

## Disclosure statement

*Financial disclosure:* The authors have nothing to disclose.

*Non-financial disclosure:* The authors have nothing to disclose.
